# *Mustn1* ablation in skeletal muscle results in increased glucose tolerance concomitant with upregulated GLUT expression in male mice

**DOI:** 10.14814/phy2.15674

**Published:** 2023-05-11

**Authors:** Charles J. Kim, Chanpreet Singh, Christine Lee, Kevin DiMagno, Madison O'Donnell, Marina Kaczmarek, Arhum Ahmed, Jessica Salvo‐Schaich, Alexis Perez, William Letsou, Maria‐Alicia Carrillo Sepulveda, Raddy L. Ramos, Michael Hadjiargyrou

**Affiliations:** ^1^ College of Osteopathic Medicine New York Institute of Technology Old Westbury New York USA; ^2^ Department of Biological and Chemical Sciences New York Institute of Technology Old Westbury New York USA

**Keywords:** glucose, GLUT, insulin, *Mustn1*, skeletal muscle

## Abstract

Glucose homeostasis is closely regulated to maintain energy requirements of vital organs and skeletal muscle plays a crucial role in this process. *Mustn1* is expressed during embryonic and postnatal skeletal muscle development and its function has been implicated in myogenic differentiation and myofusion. Whether *Mustn1* plays a role in glucose homeostasis in anyway remains largely unknown. As such, we deleted *Mustn1* in skeletal muscle using a conditional knockout (KO) mouse approach. KO mice did not reveal any specific gross phenotypic alterations in skeletal muscle. However, intraperitoneal glucose tolerance testing (IPGTT) revealed that 2‐month‐old male KO mice had significantly lower glycemia than their littermate wild type (WT) controls. These findings coincided with mRNA changes in genes known to be involved in glucose metabolism, tolerance, and insulin sensitivity; 2‐month‐old male KO mice had significantly higher expression of GLUT1 and GLUT10 transporters, MUP‐1 while OSTN expression was lower. These differences in glycemia and gene expression were statistically insignificant after 4 months. Identical experiments in female KO and WT control mice did not indicate any differences at any age. Our results suggest a link between *Mustn1* expression and glucose homeostasis during a restricted period of skeletal muscle development/maturation. While this is an observational study, Mustn1's relationship to glucose homeostasis appears to be more complex with a possible connection to other key proteins such as GLUTs, MUP‐1, and OSTN. Additionally, our data indicate temporal and sex differences. Lastly, our findings strengthen the notion that *Mustn1* plays a role in the metabolic capacity of skeletal muscle.

## 1 INTRODUCTION

Skeletal muscle represents approximately 40% of body mass and is connected to multiple regulatory processes such as blood glucose regulation and bone growth, and also functions as a paracrine and endocrine gland (Giudice & Taylor, 2017; Janssen et al., 2000). Skeletal muscle plays an important role in glucose homeostasis, consuming 70%–90% of glucose from the blood in the postprandial state. This process occurs mainly through specialized surface membrane sugar transport proteins of the solute carrier family 2 (SLC2) consisting of facilitative glucose transporters (GLUT) (Baron et al., 1988; DeFronzo et al., 1981; Mueckler, 1990; Mueckler & Thorens, 2013). Glucose is also taken up from the extracellular fluid into skeletal muscle via facilitated diffusion through GLUT proteins.

GLUT isomers display variations in their specific subcellular localization and their ability to transport different sugars (Mueckler & Thorens, 2013). During the basal (non‐insulin) stimulated state, GLUT1 is located on the cell surface while GLUT4, the most abundant glucose transporter in skeletal muscle, resides both on the cell surface and intracellularly in storage vesicles with a 1:4 ratio, respectively. When stimulated by insulin or skeletal muscle contraction, intracellular GLUT4 translocates to the cell surface of skeletal muscles to play an essential role in modulating blood glucose level (Foley et al., 2011; Jaldin‐Fincati et al., 2017; Zisman et al., 2000). Type 2 diabetes affects the ability of insulin from stimulating glucose transport in skeletal muscles by diminishing and disrupting GLUT4 translocation to the cell surface. (Garvey et al., 1998; Klip et al., 2019; Lauritzen, 2009; Ryder et al., 2000; Shepherd & Kahn, 1999; Zierath et al., 1996). Various studies have indicated that both aerobic and resistance training exercises increase GLUT4 translocation and glucose tolerance (Nishizawa et al., 2004; Shimomura et al., 2021; Yu et al., 2016).

*Mustn1* is expressed in all musculoskeletal cell types that form bone, cartilage, tendon, and muscle (Lombardo et al., 2004). The exact function of *Mustn1* remains unknown, however, its expression during skeletal muscle development in different species has been widely studied (Kim et al., 2009; Zheng et al., 2009; Liu et al., 2010; Xu et al., 2012; Gersch et al., 2012; Li et al., 2013; Xu et al., 2015; Kong et al., 2017; Camarata et al., 2016; Danzmann et al., 2016; Hadjiargyrou, 2018; Zhu et al., 2021; Wang et al., 2022). As a pan‐musculoskeletal cell marker, *Mustn1* is actively expressed in areas of early cartilage, bone, and muscle formation, specifically in limb buds, brachial arches, vertebrae, and somites during embryogenesis (Lombardo et al., 2004; Liu et al., 2010; Gersch et al., 2012). Previous studies showed that *Mustn1* is involved in bone repair as a necessary regulator for chondrocyte proliferation and differentiation, and as a positive regulator for myogenic differentiation and skeletal muscle development and function (Gersch & Hadjiargyrou, 2009; Hadjiargyrou, 2018; Krause et al., 2013; Liu et al., 2010; Lombardo et al., 2004; Nam et al., 2014). More recently, Hu et al. (2021) showed that *Mustn1* is an important molecular regulator for muscle growth and development, particularly in proliferation and differentiation of skeletal muscle satellite cells (SMSC) in chickens.

Given the important interplay between functional skeletal muscles and the metabolic capacity of the body, dysregulation of genetic networks of myogenesis could lead to impairments in skeletal muscle function. Healthy skeletal muscle can prevent and improve type 2 diabetes by increasing glucose intake and insulin sensitivity (Arora et al., 2009; Bacchi et al., 2012; Castaneda et al., 2002; Cauza et al., 2005; Hangping et al., 2019; Kadoglou et al., 2013; Sigal et al., 2007). As Mustn1 expression and function has been linked to skeletal muscle, we further investigated its role on metabolic function. Specifically, we focused on glucose tolerance and gene expression of specific skeletal muscle related genes, using a conditional knockout (KO) mouse approach. Herein, we report that *Mustn1* ablation leads to glucose tolerance increase, concomitant with upregulated GLUTs expression only in 2‐month‐old male mice.

## 2 MATERIALS AND METHODS

### 2.1 Generation of *Mustn1* conditional KO mice in skeletal muscle

**FIGURE 1:**
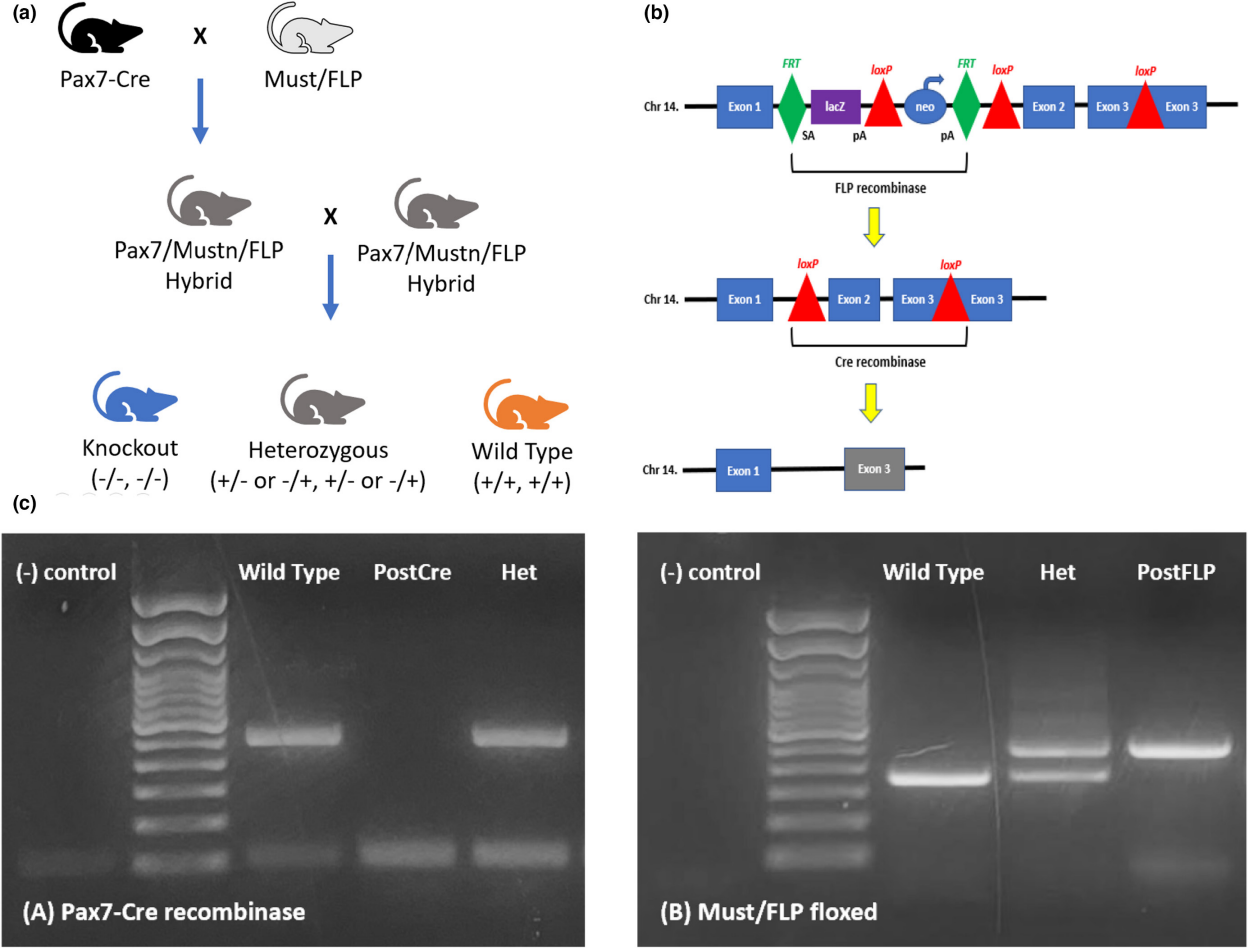
Generation of Mustn1 conditional knockout mice in skeletal muscle. (a) Breeding scheme. Initial cross between Pax‐7 Cre mouse and Must/FLP floxed mouse results in hybrid heterozygous mice, which are then crossed with each other. Homozygous Post‐FLP and Post‐Cre mice are labeled as KO and those for neither Cre or FLP, WT. The KO and WT mice were bred within the genotype to grow populations and all pups were genotyped again for verification. (b) Allele map of *Mustn1* gene. FRT marks Post‐FLP. Cre insertion encodes for Cre‐recombinase. LoxP marks the cleavage site for Cre‐recombinase enzyme. FLP recombinase cleaves between two FRT‐regions and Cre‐recombinase cleaves between two loxP regions resulting removal of exon 2 and partial of exon 3 resulting in gene inactivation. (c) Genotyping. (A) Pax7‐Cre recombinase insertion (wild type = 496 bp, Post‐Cre = 100 bp, heterozygous = 496 and 100 bp); (B) Must/FLP floxed (wild type = 356 bp, Post‐FLP = 491 bp, heterozygous = 356 and 491 bp). Mice that are homozygous Post‐Cre and homozygous Post‐FLP are labeled “KO”. Mice with homozygous of neither Post‐Cre and Post‐FLP were labeled “WT” for the control group.

All animal care and experiments were carried out in accordance with the guidelines of the Institutional Animal Care and Use Committee approved protocols. Previously, we have described the genomic organization of *Mustn1* including its promoter region (Liu & Hadjiargyrou, 2006). Generation of mice with skeletal muscle‐specific *Mustn1* inactivation was achieved by crossing pre‐existing *Mustn1* floxed mice (C57BL/6N‐A^tm1Brd^ Mustn1^tm1a(EUCOMM)Wtsi^/BayMmucd, MMRRC) with C57BL/6N‐Tg(CAG‐Flpo)1Afst/Mmucd mice (FLP). These hybrid mice were then crossed with Pax7‐Cre mice from Jackson Laboratories (C57BL/6J, Stock# 010530). The initial cross resulted in a hybrid heterozygous Pax7 +/− and Must/FLP (+/−) (Figure 1a). These hybrid mice were then crossed with each other to create an F1 generation of Pax7/Mustn/FLP mice (Figure 1a) with nine possible genotypes (Table 1). From the nine genotypes, “wild type (WT)” and “conditional knockout (KO)” labeled mice were selected as the control and experimental samples, respectively. The resulting conditional *Mustn1* KO mice have a Cre recombinase‐mediated deletion of exons 2 and part of 3 (2/3) in satellite cells and subsequent myoblasts and myocytes (Figure 1b). Two groups of mice, WT and KO, were compared specifically at 2 and 4 months to observe the effect of *Mustn1* peak expression a month before and after its natural peak expression period at 3 months (Liu et al., 2010). Mice were housed in groups of 4–5 per cage in a central specific pathogen‐free facility and kept in 12:12 h light and dark cycles with ad libitum access to standard chow and water. All animals were initially inspected for any phenotypic abnormalities and were weighed at 31, 61, 91, 120, and 180 days. Only animals (male *n* = 20; female *n* = 20) in non‐breeding same‐sex cages are analyzed to avoid confounding variables (i.e., pregnancy in females).

**TABLE 1:** Outline of possible genotypes and selection labels. Cross‐breeding Pax7/Must/FLP hybrid heterozygous mice results in nine genotypes. From these nine genotypes, mice that are homozygous Post‐Cre and homozygous Post‐FLP are labeled “Knockout” (KO). Mice with homozygous of neither Post‐Cre and Post‐FLP were labeled “Wild Type” (WT) for the control group. These selected mice were bred again within same genotypes to grow populations. All future generations were genotyped to ensure that genotypes remain consistent.

Pax7 Cre	Must/FLP	Genotype (Pax7, must/FLP)	Label
Post‐Cre	Post‐FLP	−/−, −/−	Knockout, KO (−/−)
Post‐Cre	Het	−/−, +/−	–
Post‐Cre	Wild type	−/−. +/+	–
Het	Post‐FLP	+/−, −/−	–
Het	Het	+/−, +/−	Heterozygous
Het	Wild type	+/−, +/+	–
Wild type	Post‐FLP	+/+, −/−	–
Wild type	Het	+/+, +/−	–
Wild type	Wild type	+/+, +/+	Wild type, WT (+/+)

**FIGURE 2:**
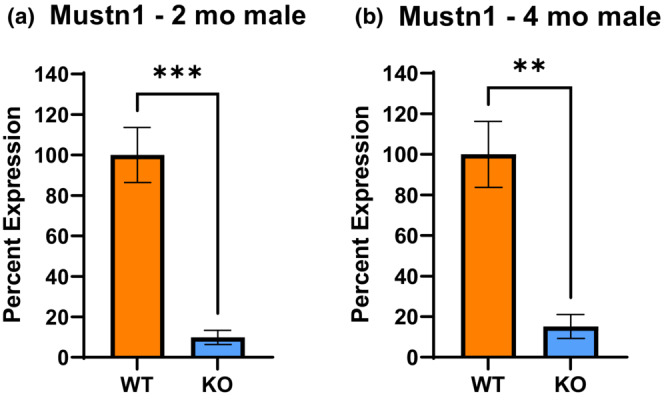
*Mustn1* mRNA expression in skeletal muscle between KO and WT male mice. *Mustn1* expression in 2‐ (a) and 4‐ (b) month‐old male mice (*n* = 4 per genotype and age **p < 0.01, ***p < 0.001, unpaired *t*‐test). Error bars indicate SEM derived from four independent PCR runs.

All mice produced by generational breeding were genotyped to confirm their genomic identity. DNA was extracted from mice tail clippings and genotyping was performed by PCR using primers (Table 2) to detect the presence of the floxed *Mustn1* and Pax7‐Cre recombinase via gel electrophoresis (Figure 1c). To assess *Mustn1* inactivation in the skeletal muscle, Quantitative real time polymerase chain reaction (Q‐PCR) was performed on WT and KO skeletal muscle cDNA with primers flanking *Mustn1* exons 2 and 3 and indeed it shows that *Mustn1* mRNA is virtually undetected in the 2‐ and 4‐month‐old KO mice (Figure 2).

**TABLE 2:** List of genes with primer sequences used for genotyping and Q‐PCR.

Target gene	Accession#	Primer sequence (5′–3′)		Amplicon size (bp)
*Mustn1*	NM 181390	Forward	CCTGAAGGCCCCATCAAGAA	123
		Reverse	GTAGTCCCGCATGACCTGG	
*FLP recombinase*	–	Forward	GGCTGAGTGAGGGAATCCTTGC	491
		Reverse	CGAGTTCAGGACTGGACACAAGG	
*Cre recombinase (WT)*	–	Forward	CTCCTCCACATTCCTTGCTC	496
		Reverse	CGGCCTTCTTCTAGGTTCTG	
*Cre recombinase (KO)*	–	Forward	GCGGTCTGGCAGTAAAAACTATC	100
		Reverse	GTGAAACAGCATTGCTGTCACTT	
*18S*	NR 003278	Forward	CGCGGTTCTATTTTGTTGGT	219
		Reverse	AGTCGGCATCGTTTATGGTC	
*GLUT1*	NM 011400	Forward	TCAACACGGCCTTCACTG	164
		Reverse	CACGATGCTCAGATAGGACATC	
*GLUT3*	NM 011401	Forward	TTCTGGTCGGAATGCTCTTC	143
		Reverse	AATGTCCTCGAAAGTCCTGC	
*GLUT4*	NM 009204	Forward	CCTCTCTTCCCTGTTACCTC	278
		Reverse	ACCCCTGCTGTTTATCCTG	
*GLUT8*	BC 090993	Forward	GGAGGCCAAGTTCAAGGACAG	419
		Reverse	CCAGAAAGGCCATGAACCAGT	
*GLUT10*	NM 130451	Forward	ACCAAAGGACAGTCTTTAGCTG	148
		Reverse	ATCTTCCAAGCAGACGGATG	
*GLUT12*	NM 178934	Forward	GGGTGTCAACCTTCTCATCTC	149
		Reverse	CCAAAGAGCATCCCTTAGTCTC	
*MUP‐1*	EU 882229	Forward	AGGATGGGAAAACCTTCCAGC	148
		Reverse	AGGCAGCGATTGGTTTTGG	
*Slc25a10*	NM 013770	Forward	TCTGGAGCAACTATGGCGTC	220
		Reverse	GGTACTCGCCCTTGGAGTTC	
*Osteocrin*	BC 117089	Forward	CTTTGGGTCTCCCCTTGACA	147
		Reverse	CATCAGCCTCTGGAACTGGAG	

### 2.2 Histology

Lower leg cross sections from male mice at 2 and 4 months were stained with hematoxylin & eosin (H & E) and Oil‐Red‐O to assess muscle histology and lipid infiltration. Tissue preparation, sectioning (8 μm), and histological examination were performed as previously reported (Cao et al., 2016; Mehlem et al., 2016). All sections were examined under a light microscope to determine the presence of any visual differences. Images were captured using a Zeiss Axio microscope attached to an Infinity 3 camera (Teledyne Lumenera).

### 2.3 Fasting glucose level

Prior to fasting, mice (*n*=10/group) were weighed and put into a clean cage individually with no food and only access to water ad libitum. Mice were fasted for 16 h in accordance with previous reports indicating higher muscle insulin sensitivity (Heijboer et al., 2005; Jensen et al., 2013). After fasting, mice were weighed again and fasting blood glucose levels (mg/dL) were measured using a glucometer (Item #8235984; Contour Next One, Parsippany, NJ) through blood samples (<5 μL) via tail vein access using aseptic techniques (Nagy & Einwallner, 2018).

### 2.4 Intraperitoneal glucose tolerance test

After 16 h of fasting and measuring the fasting blood glucose level, mice (*n* = 10/group) were anesthetized using isoflurane. Based on fasting weight, the volume of 20% glucose solution (2 g of glucose/kg body weight) was calculated. While the mouse was still under anesthesia, the glucose solution was given via intraperitoneal injection and was returned to its cage to recover. Blood glucose levels were measured at 5, 15, 30, 70, 90, and 120 min following the injection. After the final measurement of blood glucose, the mouse was returned to its original cage with food and water.

### 2.5 Intraperitoneal insulin tolerance test

After 16 h of fasting and measuring fasting blood glucose levels, the volume of insulin injection (0.75 IU/kg body weight) was calculated based on the weight post‐fasting.

The insulin (Lot #LZFV153; Novolin R, Plainsboro) was pre‐diluted to 1:400 in 0.9% NaCl (Stock: 100 U/mL insulin, working concentration 0.25 U/mL). 20% glucose was prepared to be administered if the mice became hypoglycemic. Mice (*n* = 10/group) were anesthetized using isoflurane and insulin was given via intraperitoneal injection and returned to the cage to recover after the injection. Blood glucose was measured at 3, 6, 9, 12, and 15 min after the injection through the tail vein and mice were returned to their original cage with food and water once measurements were completed.

### 2.6 Quantitative real‐time polymerase chain reaction

Under sterile conditions, right gastrocnemius muscle was excised from the hindlimb of each non‐fasted experiment‐free mouse (*n* = 4 per group) at the terminal experiments. Isolated tissue samples were immediately submerged in TRIzol™ Reagent (Invitrogen): we used 1 mL per every 50–100 mg of tissue sample, as per manufacturer's protocol. Samples were immediately homogenized using a motorized tissue homogenizer at 4°C in order to minimize sample degradation. Subsequent RNA extraction was performed following the manufacturer's standard protocol and guidelines (ThermoFisher, 2020). Following RNA extraction, samples were purified using the RNeasy Plus Mini Kit (Qiagen). Per protocol provided by the manufacturer (Qiagen), RNA quality and concentration were measured with a Thermo‐Scientific Nanodrop 2000c spectrophotometer. cDNA was synthesized from 1 μg of the extracted and purified RNA samples (template) using ReadyScript™ cDNA Synthesis Mix (Sigma‐Aldrich) and by following the manufacturer's protocol/guidelines (Sigma‐Aldrich, 2012). Yield and purity were again assessed using a Thermo‐Scientific Nanodrop 2000c spectrophotometer. RNA quality was determined by gel electrophoresis, and concentration was measured with a NanoDrop ND‐1000 (NanoDrop). Q‐PCR was carried out with LightCycler 480 SYBR Green I Master rt‐PCR kit (Roche) and the LightCycler system (Roche) following a standard protocol provided by the manufacturer (Roche, 2015). Genes of interest and their primers are listed in Table 2. Statistical significance was determined via unpaired *t*‐test.

### 2.7 Microarray analysis

The GeneChip™ WT PLUS Reagent Kit (Applied Biosystems) was used, according to manufacturer's protocol, to prepare 150 ng of total RNA (*n* = 3 for each strain) for whole transcriptome expression analysis. A hybridization cocktail containing 1.6 μg of fragmented and labeled single‐stranded DNA was hybridized to Clariom S Mouse Arrays (Applied Biosystems) for 16 h at 45°C at 60 rpm in a Genechip Hybridization Oven 640. Once the hybridization was complete, the arrays were washed and stained according to the manufacturer's protocol in a GeneChip Fluidics 450 station. The arrays were then scanned in a GeneChip Scanner 3000 G7 controlled by GeneChip Command Console v 4.3.3.1616 software. Data analysis was completed using Applied BioSystems Transcriptome Analysis Console software v4.0.2.15.

### 2.8 Bioinformatic analyses

Differential gene expression was analyzed using R version 4.2.1 and visualized in ggplot2 (Wickham, 2016). For genes with repeated probes on the array, the mean intensity and *p*‐values were taken as the representative values. For probes mapping to multiple genes, the row data were duplicated once for each unique gene in the set before taking the means. Genes were ordered by expression in the WT condition, and a fold change of two (up‐ or downregulated) in the KO condition was taken as the cutoff for differential expression.

## 3 RESULTS

### 3.1 Validation of *Mustn1* conditional knockout in skeletal muscle

Analysis of mRNA isolated from gastrocnemius muscle reveals severe downregulation of *Mustn1* mRNA in the KO mice, as expected. The KO mice displayed dramatically reduced (<20%) *Mustn1* expression at both, 2 (*p* < 0.01) and 4 months (*p* < 0.05) (Figure 2a,b), indicating successful ablation of the gene.

### 3.2 Gross morphological and histological examination

**FIGURE 3:**
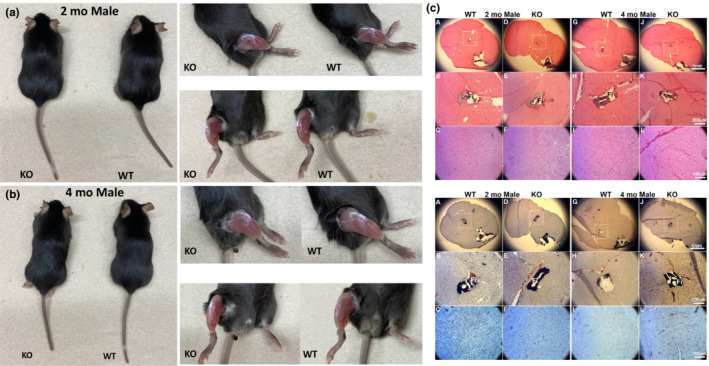
Effect of conditional knockout of *Mustn1* in skeletal muscle. Visual inspection indicated no distinct phenotypic differences between WT and KO mice. Overall appearance did not show any difference in both gender and in different age groups. Visual inspection by exposing skeletal muscle of lower limb did not show any difference. (a) Two‐month‐old male mice. (b) Four‐month‐old male mice. (c) H&E (top) and Oil‐Red‐O (bottom) staining of an 8‐μm‐thick lower leg cross section of male mice at 2 and 4 months. Dashed boxes in (A), (D), (G), and (J) are shown in higher magnification in (B), (E), (H), and K. (Fi = Fibular).

**FIGURE 4:**
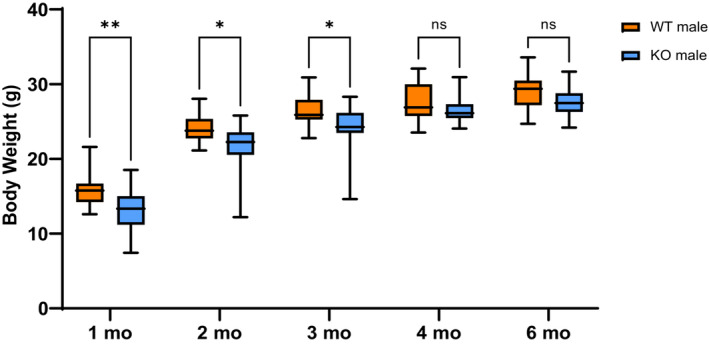
Weight measurements. Body weight of male mice per genotype at 1 month (31 days), 2 months (61 days), 3 months (91 days), 4 months (120 days), and 6 months (180 days) (*n* = 20, two‐way repeated measures ANOVA, **p < 0.01, *p < 0.05, ns = not significant.

Skeletal muscle morphology from both WT and KO mice did not exhibit any gross phenotypic differences upon visual inspection of both sexes (Figure 3a,b). Histological examination (H & E and Oil‐red) of lower leg skeletal muscle sections from male mice at 2 and 4 months also did not reveal and changes in myofiber morphology, fibrosis, or intramuscular lipid deposition, indicating that there was no significant phenotypic difference between these mice (Figure 3c). However, there was a significant weight difference in male mice from 1–3 months of age, but this difference disappeared at 4 months (although the trend was present) (Figure 4). The male KO mice weighed in average about 2.37 g less than male WT mice until 4 months, with insignificant difference in weight reduction to an average of 1.34 g from 4 months on. The female mice did not show any significant weight difference at any of the time points (Figure S1).

### 3.3 Glucose and insulin tolerance tests

**FIGURE 5:**
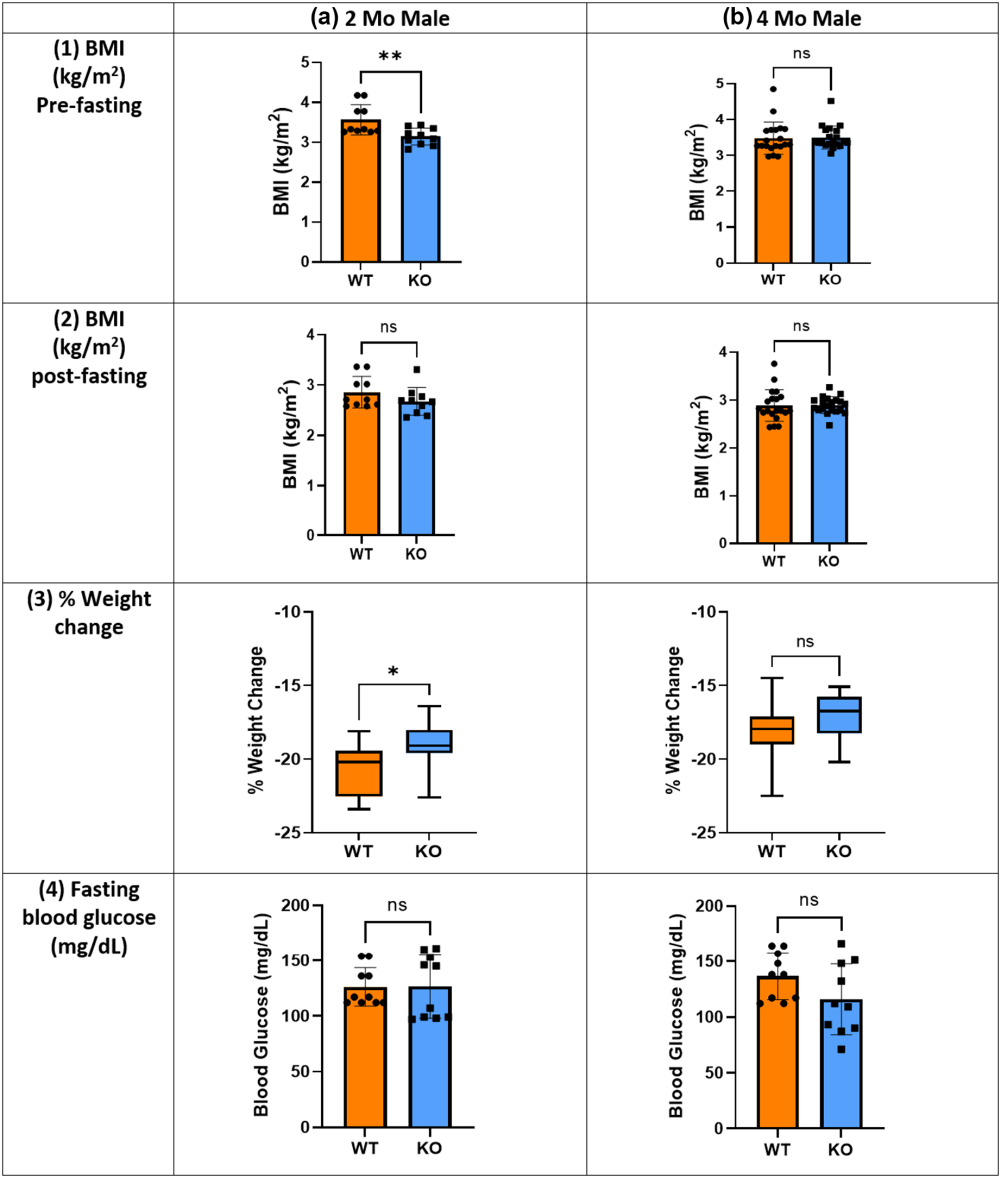
Fasting parameters and measurements. 16 h fasting (a) 2‐month‐old, (b) 4‐month‐old male mice: (1) BMI pre‐ and (2) post‐fasting; (3) percent weight change post‐fasting; (4) fasting blood glucose. *n* = 10 per genotype, *p < 0.05, **p < 0.01, Welch's *t*‐test, ns = not significant. Error bars indicate SD derived from four independent measurements.

To investigate the impact of *Mustn1* ablation on glucose homeostasis in skeletal muscle, we conducted IPGTT and ITT following 16 h of fasting. The average BMI of 2‐month‐old male mice before fasting was 3.57 ± 0.12 (mean ± SEM) for WT and 3.14 ± 0.21 for KO (*p* < 0.01) (Figure 5a), but the KO mice had significantly (*p* < 0.05) less percent weight loss than the WT, with −19.02 ± 0.52% for KO and −20.68 ± 0.55% for WT (Figure 5a). Although there is no statistically significant difference, KO mice displayed the trend of lower weight loss percentage than WT in other groups as well. However, there was no significant difference in BMI after fasting in all groups (Figure 5a,b). Lastly, there was no significant difference in fasting glucose levels (Figure 5a,b) for all age groups and sex; the 2‐month groups are similar but there was a trend of slightly lower blood glucose in the 4‐month KO male group.

**FIGURE 6:**
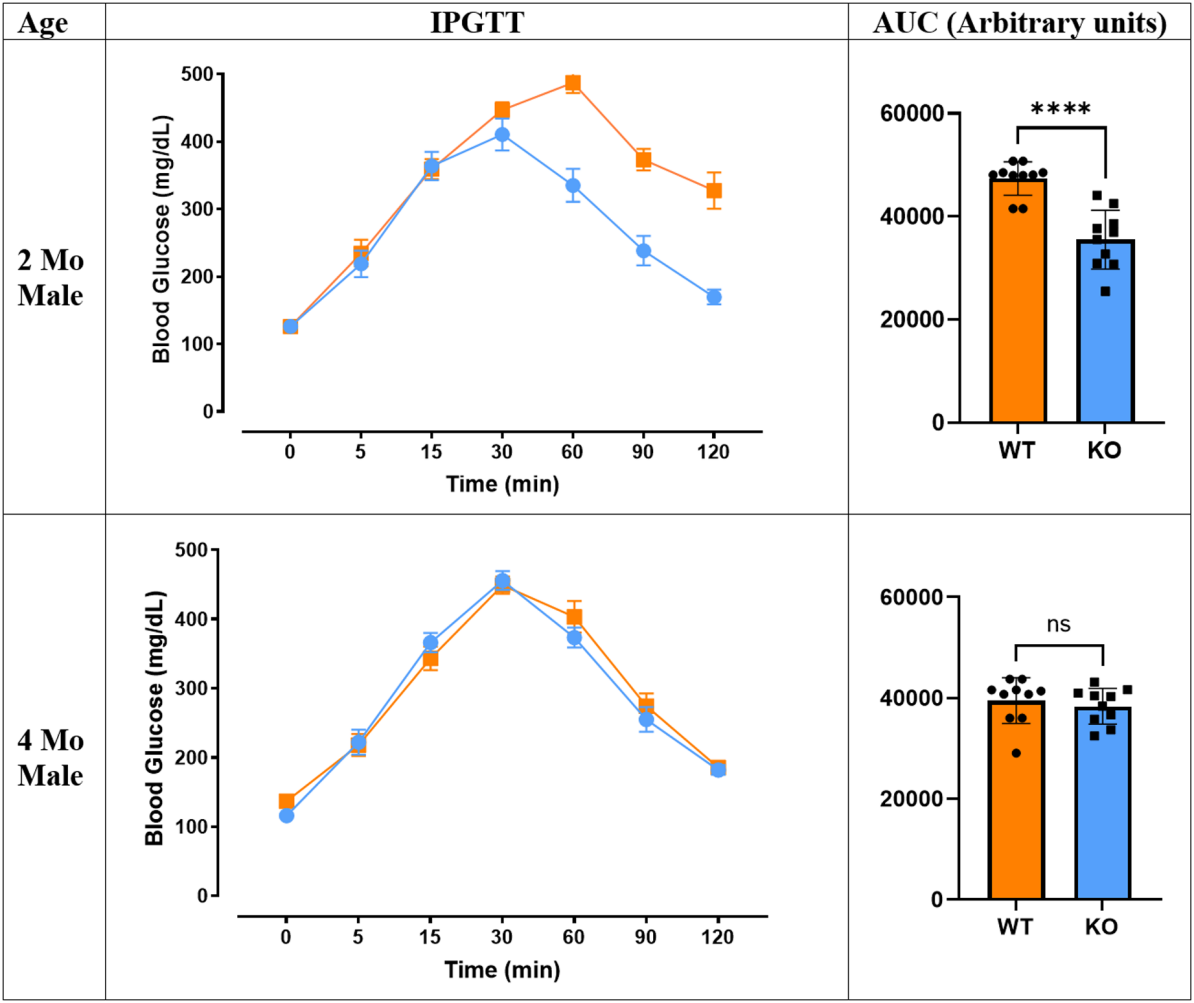
Intraperitoneal glucose tolerance test. Blood glucose levels are shown in both 2‐ and 4‐month‐old male mice over 120 min (*n* = 10, ****p < 0.0001, Welch's *t*‐test, ns = not significant). AUC is also shown in arbitrary units. Data are represented as mean ± SEM.

**FIGURE 7:**
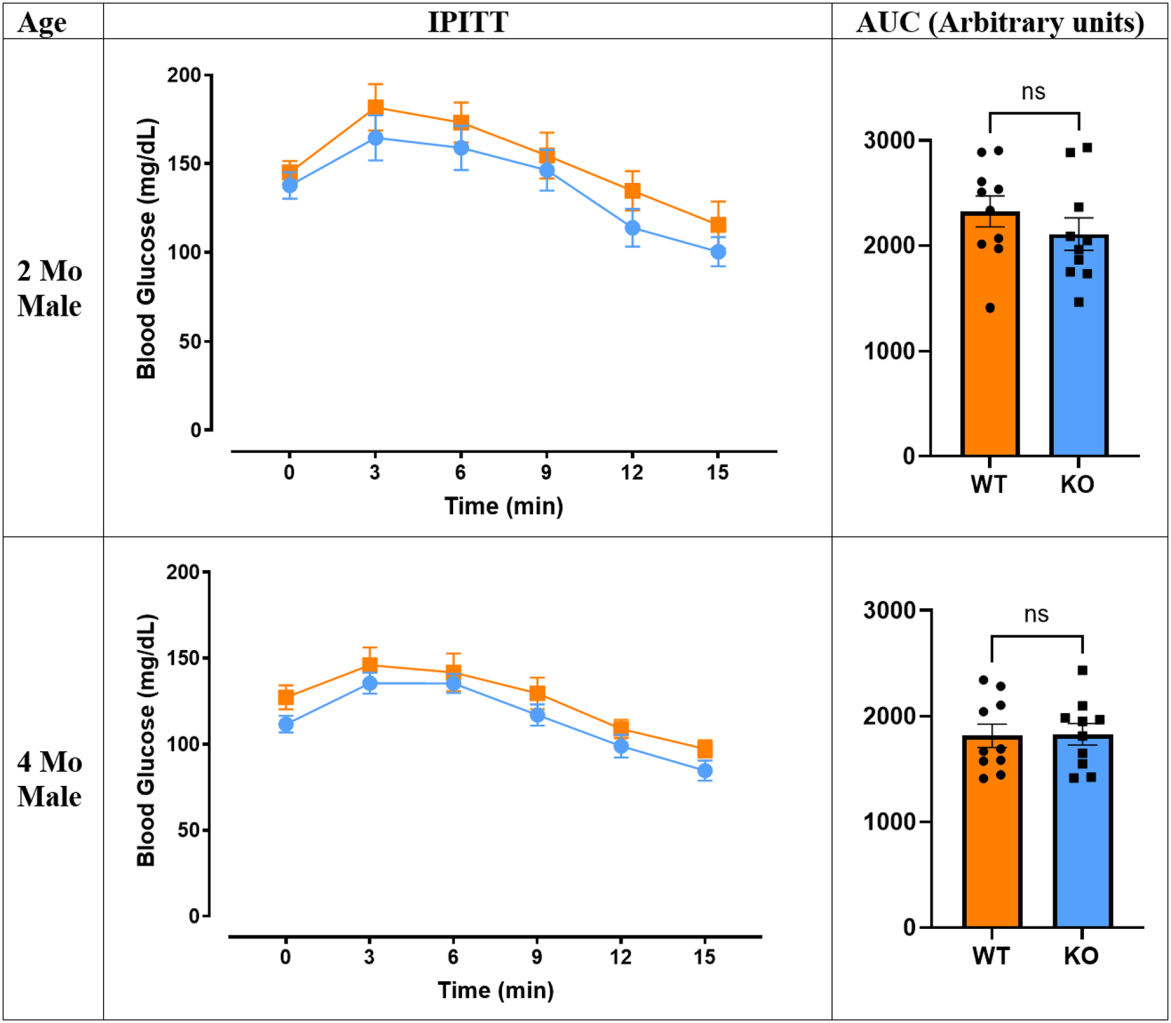
Intraperitoneal insulin tolerance test. Blood glucose levels are shown in both 2‐ and 4‐month‐old male mice over 15 min (*n* = 10 per genotype and age, Welch's *t*‐test, ns = not significant). Data are represented as mean ± SEM. AUC is also shown in arbitrary units.

The glucose tolerance test was conducted to investigate the effect of *Mustn1* ablation on glucose metabolism on skeletal muscle. The blood glucose levels were measured at 0, 5, 15, 30, 60, 90, and 120 min after glucose injection (Figure 6). The most notable difference in IPGTT was in 2‐month‐old male mice that showed significantly smaller area under the curve (AUC) in KO (*p* < 0.0001). KO and WT mice have similar fasting blood glucose level at 126.5 ± 9.01 and 126.20 ± 5.50 mg/dL, respectively, and maintain similar levels with each other for the first 15 min of the injection. The KO mice peak at 30 min with blood glucose level around 410.70 ± 23.60 mg/dL and ends at 169.90 ± 11.00 mg/dL at 120 min, while the WT mice peak at 60 min with 487 ± 15.22 mg/dL and 327.60 ± 26.83 at 120 min. This difference is not observed in the 4‐month‐old male mice. Both groups peak at 30 min with 455.70 ± 13.53 mg/dL for the KO mice and with 448.90 ± 12.57 mg/dL for the WT mice. At the 120 min, the blood glucose of the KO mice is at 181.90 ± 6.85 mg/dL and WT mice is at 185.40 ± 6.77 mg/dL, which resulted in similar AUC. The female groups did not show any differences between the genotypes in all age groups (Figure S2). Interestingly, despite the difference in glucose tolerance, the insulin tolerance test did not show any differences in all ages and between sexes (Figure 7, Figure S4).

### 3.4 Gene expression

**FIGURE 8:**
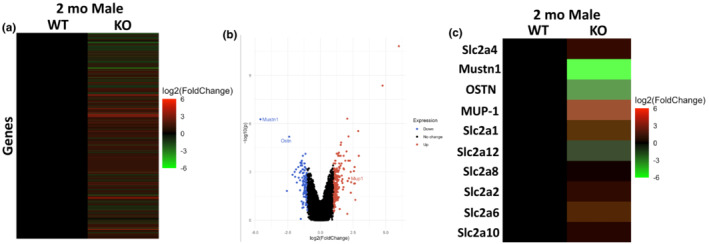
Differential mRNA expression between 2‐month‐old WT and KO male mice. **(**a) Heatmap representing fold change in expression of KO relative to WT of differentially expressed mRNAs. (b) Volcano plot of the differentially expressed mRNAs assayed. (c) Heatmap of selected genes (same analysis as shown in a). For each sample, the average of three replicates was taken and log2 transformed.

The differences in the 2‐month‐old male mice IPGTT results led us to examine possible alterations in gene expression. Thus, we employed microarray analyses (Hadjiargyrou et al., 2002; Hadjiargyrou et al., 2016) to investigate global gene expression changes between the WT and KO mice. Out of the >20,000 well‐annotated genes that were present on the microarrays, 213 genes were found to be upregulated (>2‐fold) and 93 (<2‐fold) downregulated in the KO as compared to the control WT samples. The expression signals of these differentially regulated genes are shown in a heatmap as well as a volcano plot (Figure 8a,b) and in detail in Table S1. Additionally, we present all of the genes assayed prior to the twofold cut off in Table S2. Lastly, we also show a heat map of glucose metabolism‐related genes in Figure 8c.

**FIGURE 9:**
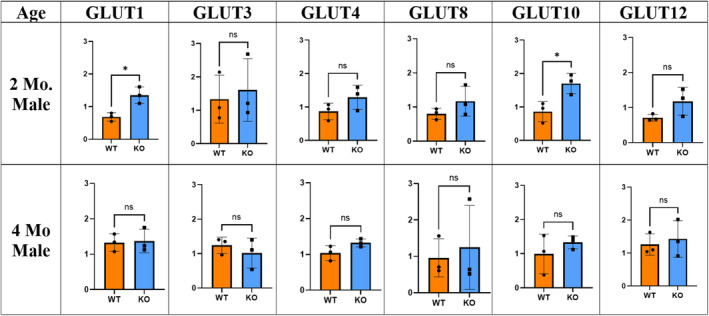
mRNA expression. Gastrocnemius expression levels of various GLUTs in 2‐ and 4‐month‐old male WT and KO mice (*n* = 4, *p < 0.05, unpaired *t*‐test, ns = not significant). Error bars indicate SD derived from three independent PCR runs.

In order to verify the microarray results shown in Figure 8c, we performed Q‐PCR and the results are presented in Figure 9. In the 2‐month‐old male cohort, expression of GLUT1 and GLUT10 was statistically greater (~1.5‐fold) in the KO mice as compared to the WT (*p* < 0.05). Although not statistically significant, other GLUT transporters (GLUT3, GLUT4, GLUT8, and GLUT12) were also elevated in the 2‐month‐old KO mice (Figure 9). This difference disappeared at 4 months similarly to the glucose tolerance test.

**FIGURE 10:**
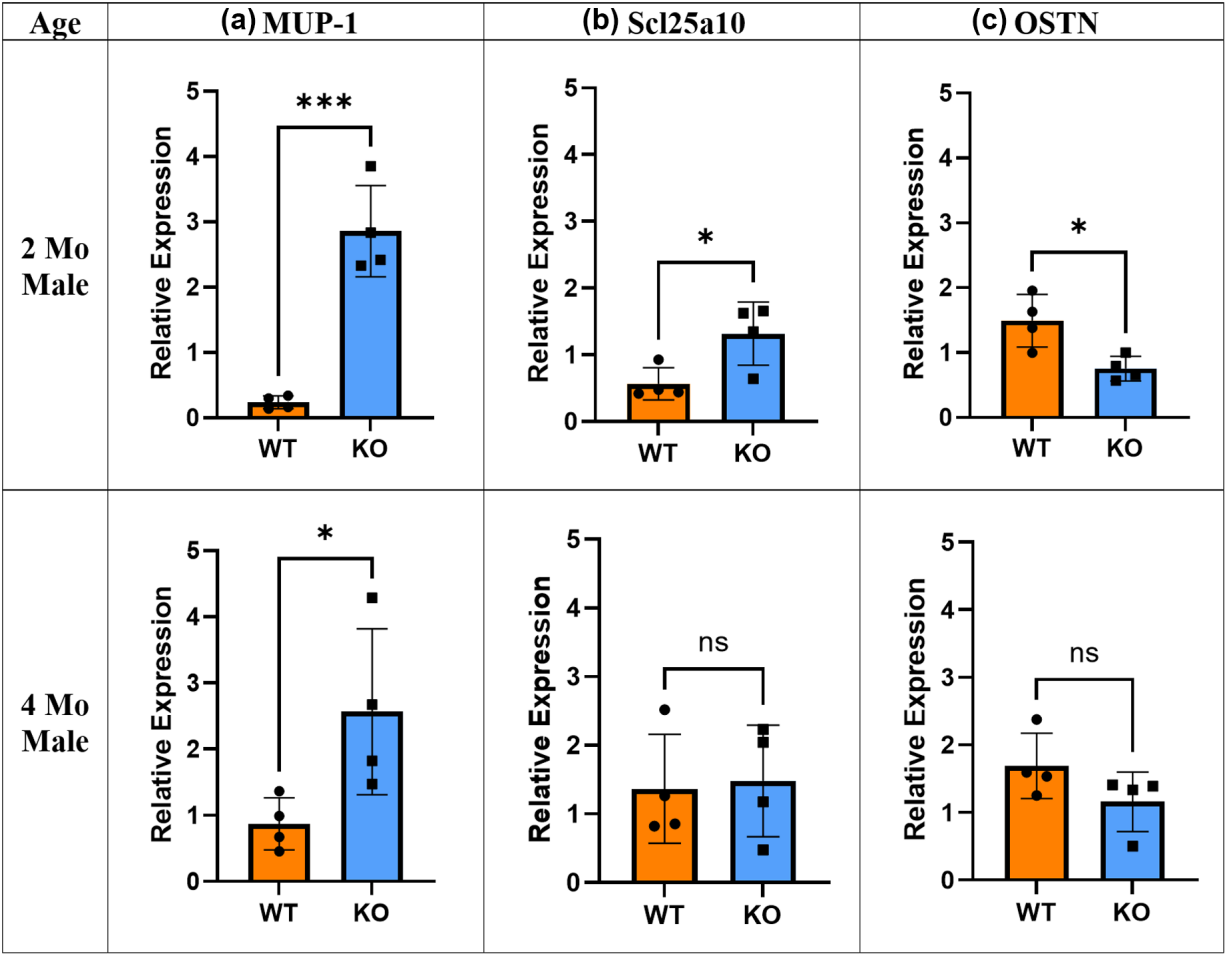
mRNA expression. Gastrocnemius expression levels of MUP‐1, Scl25a10, and OSTN in 2‐ and 4‐month‐old male WT and KO mice (*n* = 4, *p < 0.05, ***p < 0.001, Unpaired *t*‐test; ns = not significant). Error bars indicate SD derived from four independent PCR runs.

Our microarray data also showed a significant elevation of major urinary proteins (MUPs), a lipocalin family that is known to improve glucose tolerance in skeletal muscle (Hui et al., 2009) and significantly lower expression of osteocrin (OSTN), also known as musclin, a myosecretory protein that is expressed in bone and muscle and is shown to be highly upregulated in obesity‐induced insulin resistance (Nishizawa et al., 2004; Shimomura et al., 2021; Watanabe‐Takano et al., 2021; Yu et al., 2016; Zhang et al., 2021) in the 2‐month‐old KO vs. WT mice (Figure 8, Table S1). Q‐PCR was employed to verify the microarray data using MUP‐1, Scl25a10, and OSTN specific primers and RNA isolated from 2‐ and 4‐month‐old male mice. The results indicate significant increases in mRNA expression of MUP‐1 (~6‐fold) and Scla2510 (~2‐fold) while OSTN (~0.5‐fold) is significantly reduced in 2‐month‐old KO male mice. This difference also subsided at 4 months similar to other assays (Figure 10). The expression data of all genes assayed are shown in Table 3 along with their expression values from the microarray experiments and *p*‐values.

**TABLE 3:** Differential expression of specific genes. Bold indicates significance.

Gene	Fold change		Significance
*Mustn1*	Microarray	−24.08	**5.38E‐07**
	qPCR	−9.64	**0.0007**
*GLUT1*	Microarray	1.47	**0.02**
	qPCR	1.97	**0.02**
*GLUT3*	Microarray	−0.1.16	0.37
	qPCR	1.21	0.70
*GLUT4*	Microarray	1.1	0.20
	qPCR	1.49	0.16
*GLUT8*	Microarray	1.03	0.85
	qPCR	1.46	0.24
*GLUT10*	Microarray	1.07	0.59
	qPCR	1.98	**0.03**
*GLUT12*	Microarray	−1.38	0.28
	qPCR	1.66	0.11
*MUP‐1*	Microarray	6.68	**0.0006**
	qPCR	12.09	**0.0003**
*Slc25a10*	Microarray	3.32	**0.01**
	qPCR	2.33	**0.03**
*Osteocrin*	Microarray	−5.25	**6.50E‐06**
	qPCR	−0.51	**0.02**

## 4 DISCUSSION

As the first group to utilize muscle‐specific (Cre‐recombinase driven) ablation of *Mustn1* in mice in order to examine its role in glucose homeostasis, we observed a systemic effect on glucose tolerance in male mice at 2 months of age. Previously, it was reported that expression of Cre alone in beta‐cells may affect whole body glucose homeostasis (Fex et al., 2007). More importantly, it was also reported that Cre recombinase expression driven by the human α‐skeletal actin (HSA) promoter improved glucose tolerance in mice fed a high‐fat diet (Batran et al., 2018). We can rule out the effect of Cre recombinase in our experiment, since we did not generate our conditional KO mice in beta cells nor did we use the HSA promoter or fed mice a high‐fat diet. Rather, we used a murine Pax‐7 promoter to drive Cre expression in only skeletal muscle cells. Further, we also showed that female KO mice did not show any statistically significant difference in glucose tolerance, thus indicating that the presence of Cre recombinase did not affect glucose homeostasis.

All experiments were conducted on mice at 2 and 4 months of age to observe the effects of *Mustn1* ablation on skeletal muscle 1 month before and after its natural peak expression at 3 months (Liu et al., 2010). Although *Mustn1* mRNA relative expression results indicate a clear downregulation of *Mustn1* in skeletal muscle (Figure 3), the very low level of expression may be due to the possible presence of tendon left in the muscle, which is also known to express *Mustn1* (Lombardo et al., 2004). No major phenotypic changes in the organism or individual muscles were observed indicating that ablation of *Mustn1* does not severely impair the development of skeletal muscle, despite previous in vitro data indicating inhibitory effects on C2C12 myofusion and myotube formation (Liu et al., 2010) and satellite cell proliferation and differentiation (Hu et al., 2021).

Initially, we hypothesized that a *Mustn1* ablation would have impaired glucose tolerance compared to the WT, since creating a KO may disturb normal genetic networks thereby affecting normal function. Strikingly, our results revealed the opposite of what we have predicted. The C57BL/6J mouse strain is known to have a natural mutation of nicotinamide nucleotide transhydrogenase (*Nnt*), a nuclear‐encoded mitochondrial protein involved in β‐cell mitochondrial metabolism, that results in a defect of insulin secretion thus affecting glucose tolerance in male mice (Freeman et al., 2006). Our findings in the 2‐month‐old male WT mice revealed a higher blood glucose level at the end of the 2‐h glucose tolerance test, while the male KO mice in the same age group show significantly higher glucose tolerance. Interestingly, changes in the upslope of the IPGTT curve (Figure 6a, left panel) suggests an improvement on basal glucose uptake is mediating enhanced glucose tolerance observed in the male KO mice, rather than an increase in insulin secretion and sensitivity. Whereas IPGTT is a robust screening test to measure glucose uptake and assess the status of glucose tolerance as well as to estimate insulin sensitivity, IPGTT alone does not provide complete information regarding insulin secretion and sensitivity. Thus, further utilization of simple surrogate indexes for insulin resistance, such as quantitative insulin sensitivity check index (Muniyappa et al., 2008), and insulin secretion by the HOMA‐β cell index will be needed to fully characterize insulin sensitivity and secretion. Consistent with this observation, all GLUTs were expressed at a higher mRNA level in the KO mice with statistical significance for GLUT1 and GLUT10 (*p* < 0.05). At 4 months, the expression level difference dissipates between the WT and KO for all GLUTs.

Interestingly, the female cohort did not show any changes in all the experiments despite the differences observed in the male cohort. Sexual dimorphism in mouse models has previously been shown in various studies (Gao et al., 2021; Macotela et al., 2009; Tramunt et al., 2020). Our data showing an increase in MUP expression may explain the male specific effects of *Mustn1* ablation in skeletal muscle. MUP‐1 is a member of the lipocalin family that is predominantly produced in the liver, is known to act as a pheromone‐binding protein, and is secreted in the urine. There is a strong sexual dimorphism in MUP‐1 expression with ~7‐fold higher expression in males as compared to females (Hui et al., 2009). This dimorphism of MUP‐1 expression could be a possible explanation for the marked difference in glucose tolerance in 2‐month‐old male mice and no difference in female mice in the same age group. Although it is produced in the liver, its major metabolic target tissue seems to be skeletal muscle. Further, MUP‐1 increases glucose tolerance in skeletal muscle by improving mitochondrial biogenesis and Hui et al., (2009) indicated that increased glucose tolerance with MUP‐1 is achieved through increased insulin sensitivity as well as mitochondrial enhancement. However, our insulin results did not indicate any differences in insulin sensitivity in any of the groups despite significantly elevated mitochondrial solute carrier protein expression, Scl25a10, (~1.5‐fold) in the 2‐month‐old KO male mice.

The KO mice may have increased glucose tolerance through upregulated MUP‐1 and subsequent increased mitochondrial biogenesis, but insulin sensitivity is not increased even though there is high expression of GLUTs. This indicates that elevated GLUT expression alone may not affect the responsiveness of skeletal muscle cells to insulin, and thus there may be another link between GLUT and insulin. GLUT1 is predominantly localized on the muscle cell surface and is considered to be responsible for basal muscle glucose transport (Hansen et al., 1998). It was previously demonstrated that there is a positive correlation between the number of GLUT1 proteins and basal muscle glucose transport. However, the effect is not strong as ~40‐fold overexpression of GLUT1 results in only a ~9‐fold increase in basal muscle glucose transport (Hansen et al., 1998).

OSTN is another interesting secretory protein that was affected by *Mustn1* ablation in skeletal muscle. Normally, it is almost exclusively expressed in skeletal muscle and is involved with insulin sensitivity, bone growth, physical endurance, and mitochondrial biogenesis (Nishizawa et al., 2004; Re Cecconi et al., 2019; Shimomura et al., 2021; Subbotina et al., 2015; Yu et al., 2016; Zhang et al., 2021). Previous studies by Nishizawa et al. (2004) and Yu et al. (2016) indicate that overexpression of OSTN occurs with significant attenuation of insulin sensitivity, whereas exercise significantly reduces OSTN expression. Both MUP‐1 and OSTN are shown to be related to insulin sensitivity in an inverse way in type 2 diabetic rodent models (Hui et al., 2009; Shimomura et al., 2021). Interestingly, KO and WT mice exhibited major differences in glucose tolerance, however, there was no change in insulin sensitivity despite MUP‐1 and OSTN being both expressed in the direction of favoring improved insulin sensitivity in the KO model.

A general KO model (in all tissues) of *Mustn1* in mice also showed significantly improved glucose tolerance not only in males, but also in female mice (https://www.mousephenotype.org/data/genes/MGI:1913425). These general KO mice also did not have any significant phenotypic or systemic differences in glucose tolerance. The procedure was performed in the same manner as our study including 16‐h fasting period and testing at 13 weeks old (between 2 and 4 months). The fact that KO mice only had an effect on males while the general KO affected both genders could indicate that *Mustn1* has stronger effect on male skeletal muscle, hence the significant upregulation of pheromone type MUP‐1. Although we have not measured MUP‐1 in female mice, MUP‐1 expression level is known to be much lower in females (Geertzen et al., 1973; Lane & Neuhaus, 1972).

The results of our experiments suggest a possible link between *Mustn1*, MUP‐1, and OSTN that affect metabolic actions of skeletal muscle. These genes are located on different chromosomes as *Mustn1* is on chromosome 14, MUP‐1 is on chromosome 4 and OSTN is on chromosome 16 (https://www.ncbi.nlm.nih.gov/gene?Db=gene&Cmd=DetailsSearch&Term=66175; http://www.informatics.jax.org/marker/MGI:2677164; Zhou & Rui, 2010). However, there is no known relationship between these genes as of yet. The systemic effect of *Mustn1* ablation in skeletal muscle on glucose tolerance in association with transcriptional changes in GLUT, MUP‐1, and OSTN could provide further insight into glucose homeostasis. All three genes are yet to be fully understood in both function and mechanism of action and future studies are necessary in order to elucidate a further relationship between them. Additionally, one limitation of our study was that neither Cre nor the floxed allele was controlled for directly. The control mice that were used for these experiments were WT and we chose them because we believe that they serve as the “absolute” normal control group.

*Mustn1* was discovered during fracture repair study and it is thought to be playing a role in tissue repair process (Hadjiargyrou, 2018; Krause et al., 2013; Lombardo et al., 2004). This novel finding of link between *Mustn1* and GLUT could provide a more in depth understanding of how it is involved in the repair process. Decrease OSTN expression in KO mice, which is known to be involved in osteogenesis, chondrogenesis, and bone repair (Chiba et al., 2017) leads to potential future studies on the effects of conditional KO of *Mustn1* in the repair process of skeletal muscle and bone. Lastly, the downstream effects of upregulation of GLUTs in the KO mice are yet to be fully understood and the exact mechanism behind the differences between WT and KO at 4 months which dissipate remains unclear. Clearly, additional experiments should provide critical insight as to the exact mechanism by which *Mustn1* affects muscle physiology.

## Supporting information


**Figure S1.** Weight measurements. Weight of female mice per genotype at 1 month (31 days), 2 months (61 days), 3 months (91 days), 4 months (120 days), and 6 months (180 days). Female mice had no difference in weight in all age groups (*n* = 20, two‐way repeated measures ANOVA, ns = not significant).
Figure S2. Fasting parameters and measurements. 16 h fasting (A) 2‐month, (B) 4‐month‐old female mice: (1) BMI pre‐ and (2) post‐fasting; (3) Percent weight change post‐fasting; (4) Fasting blood glucose. (*n* = 10 per genotype, Welch’s *t*‐test, ns = not significant). Error bars indicate SD derived from four independent measurements.
Figure S3. Intraperitoneal glucose tolerance test blood glucose levels are shown in both 2‐ and 4‐month‐old female mice over 120 min (*n*=10, Welch’s *t*‐test, ns = not significant). Data represented by mean ± SEM. AUC is also shown in arbitrary units.
Figure S4. Intraperitoneal insulin tolerance test. Blood glucose levels are shown in both 2‐ and 4‐month‐old female mice over 15 min (*n* = 10 per genotype and age, Welch’s *t*‐test, not significant). Data represented by mean ± SEM. AUC is also shown in arbitrary units.

**Table S1.** Genes with a < or >2‐fold level as differentially expressed between KO and WT mice.

**Table S2.** Differentially expressed genes between KO and WT samples.
