# Supplementary material for: Mustn1 ablation in skeletal muscle results in increased glucose tolerance concomitant with upregulated GLUT expression in male mice
Source: Physiol Rep. 2023 May 11;11(9):e15674. doi: 10.14814/phy2.15674 (PMC10175242; doi:10.14814/phy2.15674)
Supplement: Supplementary file 1 — Figure S1. Weight measurements. Weight of female mice per genotype at 1 month (31 days), 2 months (61 days), 3 months (91 days), 4 months (120 days), and 6 months (180 days). Female mice had no difference in weight in all age groups (n = 20, two‐way repeated measures ANOVA, ns = not significant). Figure S2. Fasting parameters and measurements. 16 h fasting (A) 2‐month, (B) 4‐month‐old female mice: (1) BMI pre‐ and (2) post‐fasting; (3) Percent weight change post‐fasting; (4) Fasting blood glucose. (n = 10 per genotype, Welch’s t‐test, ns = not significant). Error bars indicate SD derived from four independent measurements. Figure S3. Intraperitoneal glucose tolerance test blood glucose levels are shown in both 2‐ and 4‐month‐old female mice over 120 min (n=10, Welch’s t‐test, ns = not significant). Data represented by mean ± SEM. AUC is also shown in arbitrary units. Figure S4. Intraperitoneal insulin tolerance test. Blood glucose levels are shown in both 2‐ and 4‐month‐old female mice over 15 min (n = 10 per genotype and age, Welch’s t‐test, not significant). Data represented by mean ± SEM. AUC is also shown in arbitrary units. [file PHY2-11-e15674-s003.pdf]

Supplementary Data

Figure 1. Weight measurements. Weight of female mice per genotype at 1 month (31 days), 2 months (61 days), 3 months (91 days), 4 months (120 days) and 6 months (180 days). Female mice had no difference in weight in all age groups (n = 20, Two-way Repeated Measured ANOVA, ns = not significant).

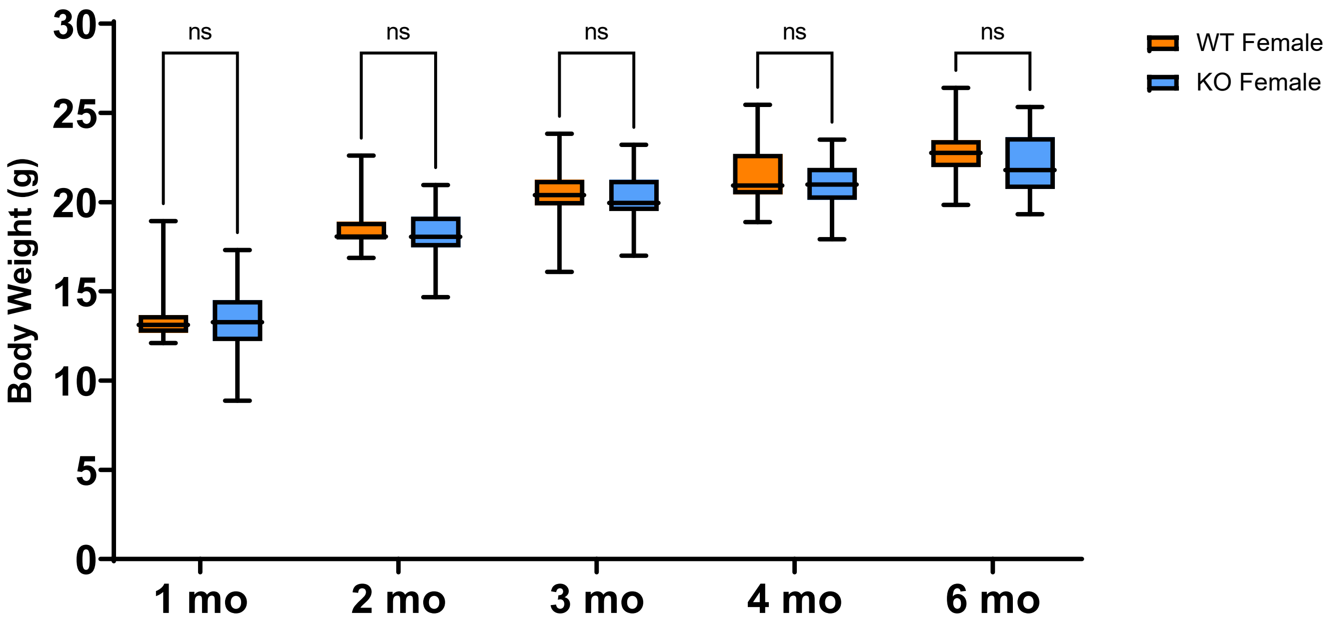

Figure 2. Fasting parameters and measurements. 16 hour fasting (A) 2-months, (B) 4-months female mice: (1) BMI pre- and (2)post-fasting; (3) Percent weight change post-fasting; (4) Fasting blood glucose. (n = 10 per genotype, Welch's t-test, ns = not significant). Error bars indicate SD derived from four independent measurements.

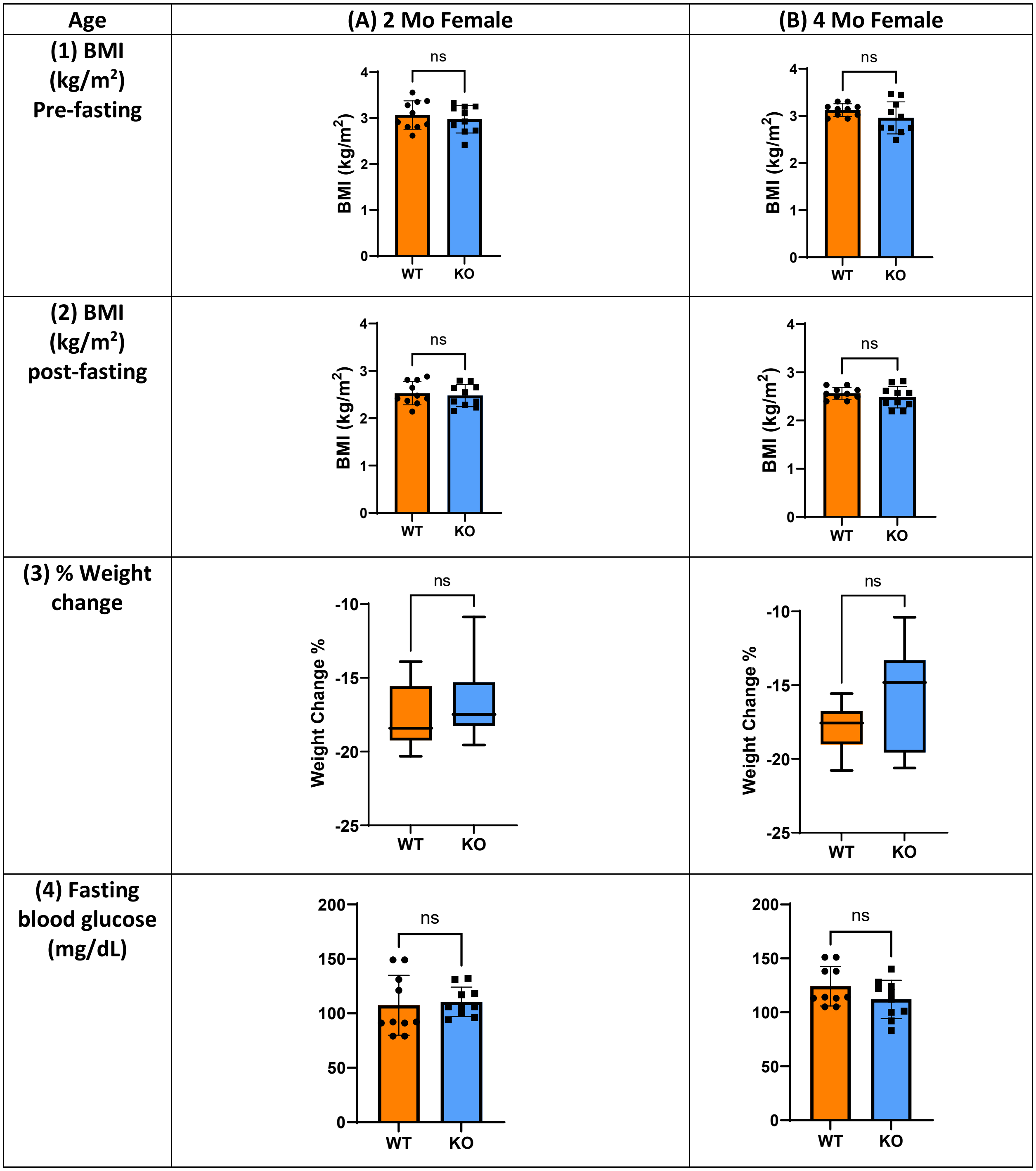

Figure 3. Intraperitoneal Glucose Tolerance Test Blood glucose levels are shown in both 2 and 4 month female mice over 120 min (n=10, Welch's t-test, ns = not significant). Data represented by mean ± SEM. AUC is also shown in arbitrary units.

<table>
<tr><th>Age</th><th>IPGTT</th><th>AUC (Arbitrary units)</th></tr>
<tr><td>2 Mo Female</td><td>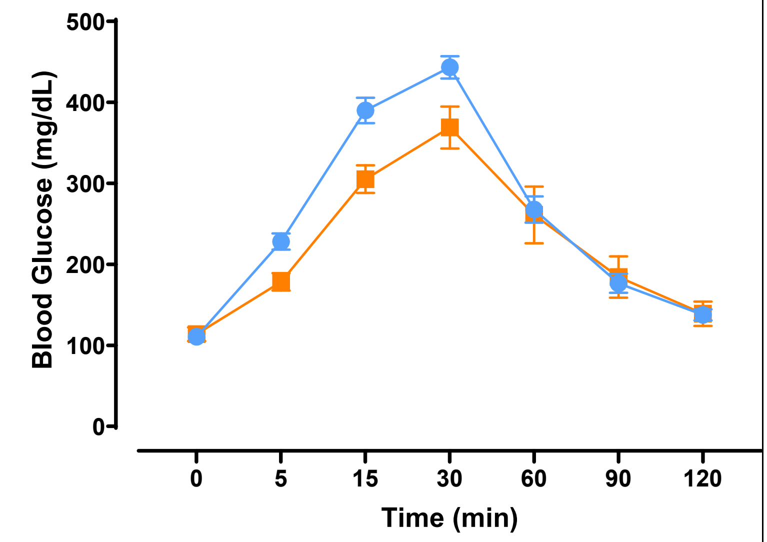
</td><td>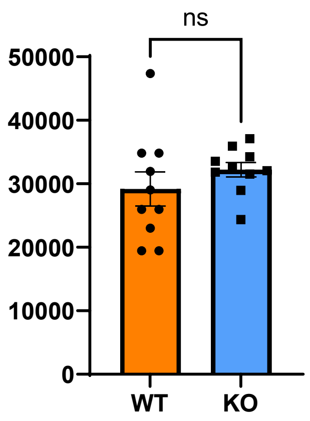
</td></tr>
<tr><td>4 Mo Female</td><td>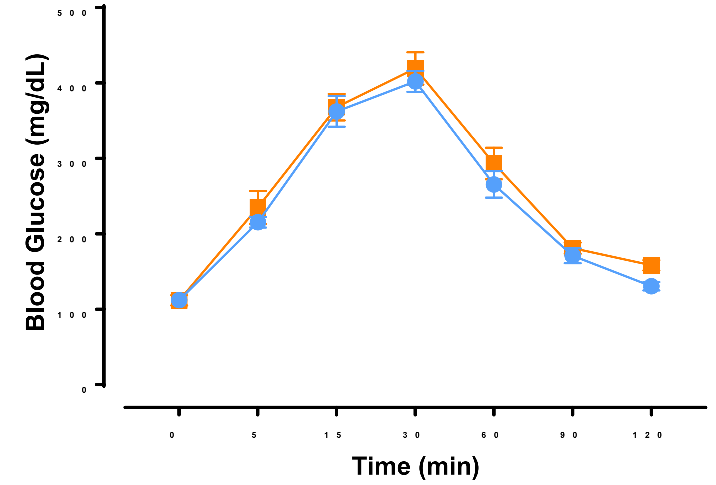
</td><td>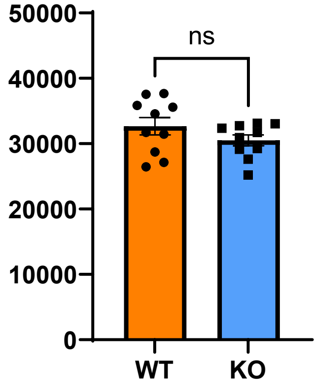
</td></tr>
</table>

Figure 4. Intraperitoneal Insulin Tolerance Test. Blood glucose levels are shown in both 2 and 4 month female mice over 15 min (n=10 per genotype and age, Welch's t-test, not significant). Data represented by mean ± SEM. AUC is also shown in arbitrary units.

Age	IPITT	AUC (Arbitrary units)
**2 Mo Female**	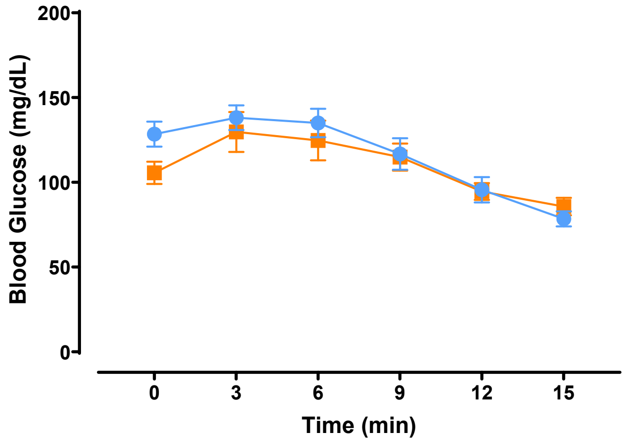	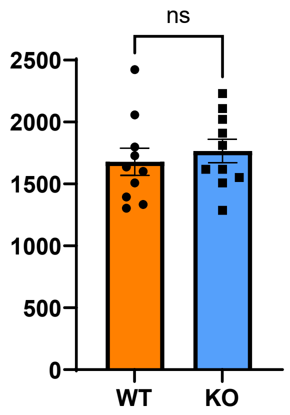
**4 Mo Female**	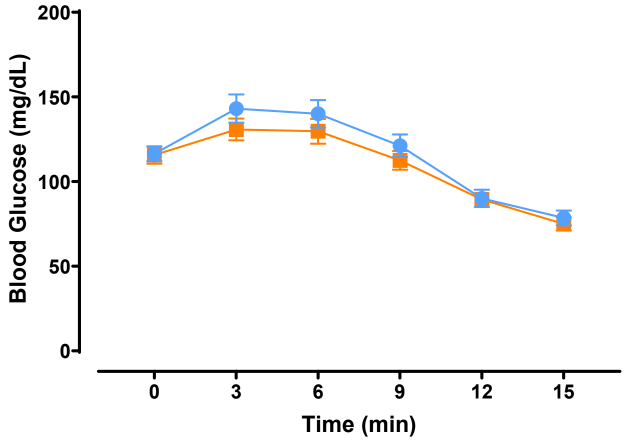	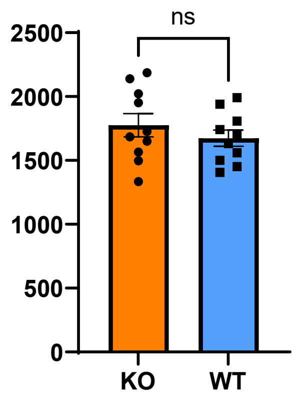
